# Genetic diversity, linkage disequilibrium, and association mapping analyses of *Gossypium barbadense* L. germplasm

**DOI:** 10.1371/journal.pone.0188125

**Published:** 2017-11-14

**Authors:** Alisher A. Abdullaev, Ilkhom B. Salakhutdinov, Sharof S. Egamberdiev, Ernest E. Khurshut, Sofiya M. Rizaeva, Mauricio Ulloa, Ibrokhim Y. Abdurakhmonov

**Affiliations:** 1 Center of Genomics and Bioinformatics, Academy of Sciences of Uzbekistan, Tashkent, Uzbekistan; 2 Institute of Genetics and Plant Experimental Biology, Academy of Sciences of Uzbekistan, Tashkent, Uzbekistan; 3 Cropping Systems Research Laboratory, United States Department of Agriculture - Agricultural Research Services, Lubbock, Texas, United States of America; New South Wales Department of Primary Industries, AUSTRALIA

## Abstract

Limited polymorphism and narrow genetic base, due to genetic bottleneck through historic domestication, highlight a need for comprehensive characterization and utilization of existing genetic diversity in cotton germplasm collections. In this study, 288 worldwide *Gossypium barbadense* L. cotton germplasm accessions were evaluated in two diverse environments (Uzbekistan and USA). These accessions were assessed for genetic diversity, population structure, linkage disequilibrium (LD), and LD-based association mapping (AM) of fiber quality traits using 108 genome-wide simple sequence repeat (SSR) markers. Analyses revealed structured population characteristics and a high level of intra-variability (67.2%) and moderate interpopulation differentiation (32.8%). Eight percent and 4.3% of markers revealed LD in the genome of the *G*. *barbadense* at critical values of r^2^ ≥ 0.1 and r^2^ ≥ 0.2, respectively. The LD decay was on average 24.8 cM at the threshold of r^2^ ≥ 0.05. LD retained on average distance of 3.36 cM at the threshold of r^2^ ≥ 0.1. Based on the phenotypic evaluations in the two diverse environments, 100 marker loci revealed a strong association with major fiber quality traits using mixed linear model (MLM) based association mapping approach. Fourteen marker loci were found to be consistent with previously identified quantitative trait loci (QTLs), and 86 were found to be new unreported marker loci. Our results provide insights into the breeding history and genetic relationship of *G*. *barbadense* germplasm and should be helpful for the improvement of cotton cultivars using molecular breeding and omics-based technologies.

## Introduction

Cultivated cotton (*Gossypium* spp.) is the most important natural fiber worldwide. Fiber quality is a key factor for determining price and quality of cotton textile products, and is significantly affected by different environmental factors [1]. In addition, genetic improvement of fiber quality is a challenge due to the narrow genetic base of modern cotton cultivars and the existence of negative correlations between major fiber quality traits and key agronomic characteristics [2, 3]. All of the above highlight a great need to study genetic resources preserved and maintained in world cotton germplasm collections [4], and to use these resources in breeding of superior cotton genotypes.

There are several major cotton germplasm collections in the world. One of the biggest and richest germplasm collections is housed in Uzbekistan with extensive genetic diversity [4, 5]. In the *Gossypium* genus, genetic diversity exists in its genome with unique traits or sometimes hidden elements, or genes that can have a positive impact on the expression of agronomic traits and resistance to biotic and abiotic factors. Introduction of valuable traits in modern cotton germplasm enriches and improves the diversity of cultivated cotton [4–8]. Genetic studies and evaluations of cotton germplasm resources provide specific information on the degree of phylogenetic relatedness of accessions in these collections and its/their representation. In addition, evaluations shed light on many questions of complex genetic traits that will eventually allow the use of the genetic potential of cotton germplasm for introduction of important and useful features/traits in modern cotton cultivars. For the introduction of important traits, marker-assisted selection (MAS) is one of the key and valuable tools for the introduction and introgression of useful traits.

Unfortunately, MAS in cotton lags behind other crops due to limited genetic polymorphism of cultivated cotton germplasm as a result of the historical process of domestication [9–11]. This also complicates the process of genetic mapping of quantitative trait loci (QTLs) associations with traits of interest using DNA markers. Moreover, much of the molecular-genomic researches including association studies and MAS focused on members of the species *G*. *hirsutum* [12–20]. This species supplies around 95.5% of the cotton production worldwide. Studies in other species unfortunately are limited as is the case with *G*. *barbadense* germplasm resources [21].

The *G*. *hirsutum* (also known as Upland cotton) and *G*. *barbadense* [also known as Sea Island, Egyptian, or extra-long staple (ELS) cotton] are the two main widely grown cotton species. Although *G*. *barbadense* only accounts for around 4.5% of the cotton production worldwide, this species is known for its superior fiber quality (length, fineness and strength). Its fiber is highly valued in the premium textile market [22, 23]. *G*. *barbadense* is indigenous to the northern part of South America and extends into Mesoamerica and the Caribbean [24]. In the United States, modern elite *G*. *barbadense* cultivars trace their origins to the Sea Island cottons developed on the coastal islands of Georgia and South Carolina that probably originated from west Andean Peruvian germplasm [24, 25]. The Sea Island cotton production collapsed in the USA under boll weevil (*Anthonomus grandis* Boheman) pressure in 1920 [7]. This Sea Island pool contributed to the development of the Egyptian cottons which in the 1940s were reintroduced into the USA as a part of the genetic base of the Pima gene pool by USDA-ARS [22, 24].

Molecular marker technology and QTL-mapping approach using bi-parental mapping populations resulted in a number of potential DNA markers for future breeding programs of cotton through MAS [11]. A classic QTL-mapping using bi-parental population exploits the short history of recombination and consequently QTL can only be localized to large chromosomal regions. By contrast, mapping approaches that exploit linkage disequilibrium make use of all recombination that have occurred during the breeding history resulting in much higher mapping resolution [26, 27]. The extent of genome-wide LD or allelic association is the key starting point for association mapping (AM). Quantification of the LD extent and AM have been successfully applied for many plant species [28, 29] including cotton [9, 30–33]. Recent LD-based studies of *G*. *hirsutum* germplasm resulted in association mapping of *Verticillium* wilt resistance [18], salinity tolerance [31] and seed oil and protein contents [32].

Here, we for the first time report SSR marker-based genetic analyses of 288 *G*. *barbadense* germplasm accessions of Uzbekistan germplasm collection, grown and phenotypically evaluated in two diverse environments, Uzbekistan and USA. The molecular genetic characteristics and diversity, population structure, the extent of LD, and association mapping of main fiber quality traits of *G*. *barbadense* germplasm are reported based on genotypic data of a core set of 108 microsatellite markers, evenly distributed in the cotton genome. SSR marker loci were statistically associated with fiber quality traits specific to Uzbekistan and USA environments. Mixed linear model (MLM) analysis, considering confounding effects of structured population, detected 100 reliable SSRs, which were common and statistically significant in the two distinct environments, Uzbekistan and USA. The results of this study are, to the best to our knowledge, the first report on a genome-wide LD analysis and LD-based AM of fiber quality traits using SSR markers in *G*. *barbadense* germplasm resources of Uzbekistan. In addition, these findings are very useful for the application of association study in cotton and should accelerate the development of superior cotton cultivars through MAS programs.

## Materials and methods

### Plant materials

The Uzbek cotton germplasm collection houses and preserves more than 7500 accessions of different cotton species at the Institute of Genetics and Plant Experimental Biology (IG&PEB), Academy of Sciences of Uzbekistan. Out of 7500 cotton germplasm accessions, *G*. *barbadense* comprises approximately 13% with broad geographic and breeding coverage. A total of 288 *G*. *barbadense* germplasm accessions from Central Asian (237), African (35) and American (16) origin were selected from the Uzbek collection and used in this study, including for genome-wide LD and association mapping analyses.

### Phenotypic analyses in the Uzbekistan environment

Analyses of morpho-biological characteristics of these selected *G*. *barbadense* germplasm and cultivars were performed in the field stations of the IG&PEB, Tashkent, Uzbekistan in 2010. Standard field plots, irrigation and agronomic technologies were used for growing cultivars in the Tashkent cultivation environment. Detailed information about weather data for specific years can be obtained from the archive of the meteorology center (http://www.wunderground.com). Ten plants of each cultivar were grown and self-pollinated by sealing flowers with florist wire just before the flowers opened. Cotton fiber samples from self-pollinated cotton bolls were harvested from field-grown plants in the beginning of October. At least 25 fully opened self-pollinated cotton bolls were harvested from each group of cultivars (pooled from ten plants per accession). Fiber quality traits of cultivars grown in Uzbekistan environment, such as fiber length (FL), fiber strength (FS), fiber micronaire (FM), and fiber uniformity (FU), were measured for 247 accessions (Table 1; S1 Data) by High Volume Instrument (HVI) of the certified “SIFAT” agency, Tashkent, Uzbekistan.

**Table 1:** Descriptive statistics of fiber traits among *G*. *barbadense* accessions grown in the Uzbekistan and USA environments.

Traits	No. of samples¶	Mean	Min.	25 percentile	Median	75 percentile	Max.	10 percentile	90 percentile	SD	CV
**Tashkent, Uzbekistan**											
**Micronaire**†	247	4.25	3.0	3.9	4.3	4.6	6.3	3.6	4.9	0.51	12.22
**Strength**	247	38.81	26.4	37.4	38.8	40.4	49.3	35.7	42.3	2.95	7.62
Length*	247	1.30	0.95	1.26	1.32	1.36	1.5	1.19	1.4	0.08	6.51
Uniformity**	247	84.74	78.9	83.8	84.8	85.9	88.6	82.7	86.6	1.60	1.89
**California, USA**											
**Micronaire**	278	4.06	2.6	3.7	4.1	4.4	5.3	3.4	4.6	0.46	11.45
**Strength**	278	36.50	26.7	34.9	36.4	38.1	43.7	33.6	40.1	2.61	7.16
**Length**	278	1.36	1.0	1.32	1.36	1.41	1.58	1.26	1.45	0.08	6.04
**Uniformity**	278	87.30	81.6	86.5	87.5	88.3	90.0	85.5	89.0	1.42	1.63

^¶^Samples evaluated in Uzbekistan and the USA represented a set of initially chosen 288 *G*. *barbadense* accessions;

^†^Characteristics and fineness of the cotton fiber maturity determined by the air permeability of the fiber sample, expressed as an index;

*Length of HVI according to the standards specified in inches (1 inch = 25.4 mm);

** Percentage of the average fiber length to the average upper length

### Phenotypic analyses in the California environment

In 2010, the germplasm and cultivars were evaluated under the San Joaquin Valley environment of California, Shafter Research Station (35°31’52” N 119°16’41” W), Kern County, USA. Accessions were grown in one-row plot, 5 by 1 meter, in a complete randomized design with a plant density ranging from 40 to 50 plants per plot. To examine fiber quality traits, 50 open matured bolls were randomly harvested from different segments of the plant from different plants of each plot. After processing using a saw gin, samples were sent to the USDA Cotton Classing Laboratory, Visalia, CA for analyses. Fiber length (FL) in mm, fiber strength (FS) in kN m kg^-1^, fiber micronaire (FM), and fiber uniformity (FU) in percentage were measured for 278 accessions (Table 1, S1 Data) by High Volume Instrument (HVI).

Due to use of wild-type genotypes of plant material, available in *ex situ* germplasm collection and commonly used for research, no authority permission was required to evaluate cotton germplasm resources in Uzbekistan and the USA. Plant material evaluated was not under the control of relevant regulatory bodies concerned with wildlife protection, and the field studies did not involve endangered or protected species. All field evaluations were conducted in the experimental stations, a priori assigned for research activities, which did not require a specific permission for conducting field evaluations.

### Statistical analyses

Data were analyzed using analysis of variance (ANOVA) [34] for the different fiber values, and correlations analyses were performed to examine the similarity of value-responses of the different accessions and fiber traits in the two growing-environments, Uzbekistan and California.

### SSR analysis

In total, 750 SSR primer pairs from different SSR collections were screened to detect polymorphisms among accessions. Genomic DNAs were isolated from leaf tissues of each germplasm or cultivar using the method of Dellaporta et al. [35]. Each accession was genotyped using 108 polymorphic SSR marker primers distributed an average of 4 SSR markers per each cotton chromosome. SSRs were chosen based on previous germplasm collection characterizations [9, 33], and based on information related to important QTLs and chromosome distribution.

Polymerase chain reaction (PCR) mixtures (10 μL) consisted of 1X reaction buffer, 1.5 mM MgCl2, 0.2 mM dNTP, 0.3 μM primers, 25 ng template DNA, and 0.5 U Taq DNA polymerase (Applied Biosystems, Foster city, USA). Amplification was carried out in a GeneAmp 9700 (Applied Biosystems), with an initial denaturation at 95°C for 10 min, followed by 35 cycles of 1 min at 94°C, 1 min at X°C, and 1 min at 72°C, plus a 5-min final extension at 72°C. X°C refers to the annealing temperature specified for each primer. The amplified products were separated on 3% (w/v) high resolution agarose gels (GeneMate, Radnor, USA) and visualized under UV light with ethidium bromide staining.

### Genetic diversity and phylogenetic analyses

The amplified fragments of each SSR marker were scored based on fragment sizes (S2 Data). Polymorphism information content (PIC) of SSR markers was calculated using the PowerMarker software package [36]. The heterozygosity level of marker data was identified according to an average similarity frequency of alternative alleles [37]. Allele frequencies were calculated using SpaGeDi software [38]. Genetic distance and phylogenetic analyses of cotton cultivars were performed using Neighbor Joining (N-J) algorithms in PAUP*4.0 [39]. Genetic variation within and among predefined groups and pair-wise F_ST_ genetic distances were measured by Analysis of Molecular Variance (AMOVA) [40–42] using ARLEQUIN 2.0 [43]. A Bayesian partition method of genetic differentiation among population groups was applied using HICKORY [44] software to direct estimation of F_ST_ without prior knowledge of inbreeding history [45].

### Population structure and kinship analyses

The population structure of the 288 *G*. *barbadense* germplasm and cultivars was assessed using the model-based (Bayesian clustering) method implemented in STRUCTURE v2.3.3 [46]. The number of subgroups (K) was set from 1 to 12 based on models characterized by admixture and correlated allele frequencies. For each K, ten runs were performed separately, with 100,000 iterations carried out for each run after a burn-in period of 10,000 iterations. The true number of sub-populations was estimated using the method proposed by Evanno et al. [47] Accessions were assigned to a subgroup if the probability of membership was greater than 70% [48]. A pairwise kinship (K-matrix) estimate for 288 *G*. *barbadense* accessions was calculated according to Hardy [49] using the software package SpaGeDi [38].

### Pair-wise linkage disequilibrium and LD decay

The genome-wide LD between pairs of SSR marker loci in the *G*. *barbadense* genome was studied according to Whitt and Buckler [48] using the software package TASSEL [50]. SSR alleles with a 0.05 frequency in genotyped accessions were removed before conducting LD analyses because minor alleles are usually problematic and biased for LD estimates between pairs of loci [51, 52]. LD was estimated by a weighted average of squared allele-frequency correlations (r^2^) between SSR loci. Loci were considered to be significant at p-values≤0.005 among all possible SSR loci. LD was evaluated with the rapid permutation test in 10,000 shuffles. Values of LD between all pairs of SSR loci were plotted as triangle LD plots using TASSEL to estimate the general view of genome-wide LD patterns and evaluate ‘block-like’ LD structures. The r^2^ values for pairs of SSR loci were plotted as a function of map distances (cM), and LD decay (at r^2^ >0.1) was estimated [48].

### Association mapping of fiber quality traits

Association mapping using the mixed linear model (MLM) and general linear models (GLM) was performed for both environments and for the four major fiber quality traits data [fiber length (FL), fiber strength (FS), fiber uniformity (FU), and fiber micronaire (FM); S1 Data]. To construct marker-fiber quality trait associations using SSR and fiber data (S1 and S2 Data), the MLM test was performed according to Yu et al. [53] using the TASSEL software package [50]. The MLM association test was simultaneously performed by accounting of multiple levels of population structure (Q-matrix) and relative kinship among the individuals (K-matrix) [50–55].

The 5% of ‘minor alleles’ filtered-SSR datasets were used for all association mapping models. Fiber trait data was imputed for missing data and normalized using algorithms implemented in TASSEL before conducting an association mapping analysis. The MLM-derived p-values were separately tested for multiple testing correction using pFDR test in QVALUE program version 1.0 [56], Sidak procedure of Bonferroni adjustment, and pACT method of Conneely and Boehnke [57]. To reliably interpret the MLM-derived significant associations, a minimum Bayes factor (BFmin) was calculated using the following formula:BFmin = -e*p*ln(p) [33, 58, 59–61]. Moreover, the MLM-derived significant associations were also subjected to comparisons with published literature information to judge obtained associations.

## Results

### Fiber quality properties of *G*. *barbadense* cultivars in the USA and Uzbekistan environments

Due to missing research plots of some accessions and/or technical errors during HVI analyses, major fiber trait measurements for 247 and 278 accessions were obtained in the Uzbekistan and USA environments, respectively (Table 1). Herein, we reference 288 accessions as a total number for the molecular set panel investigated in these two environments. A wide-range of phenotypic variation in fiber quality traits such as FL, FM, FU, FS was observed in both environments (Table 1).

The coefficient of experimental variability of traits in California (USA) and Tashkent (Uzbekistan) conditions ranged from as low as 1.63–1.89 (FU) and as high as 11.45–12.22 (FM) for the above traits. Thus, micronaire was the most variable (2.6 to 6.3) trait of all the fiber quality parameters and showed similar values of coefficient of variation in both environments. The lower variations of micronaire values (2.6 to 3.4) were observed in California’s environment, while in Tashkent, values shifted toward the high (from 4.6 to 6.3) values.

Fiber strength for all *G*. *barbadense* accessions in the Tashkent environment ranged from 270.0 to 503.5 kN m kg^-1^ (27.0 g/tex^-1^ to 50.35 g/tex^-1^) with an average value of 396.0 kN m kg^-1^ (39.6 g/tex^-1^; SD = 29.6). In California, the FS had minimum and maximum values equal to 273.0 and 446.0 kN m kg^-1^ (27.3 g/tex^-1^ to 44.6 g/tex^-1^) respectively, with an average of 373.0 kN m kg^-1^ (37.3 g/tex^-1^; SD = 26.1).

Moreover, comparisons of fiber traits among *G*. *barbadense* accessions from the four major geographical groups (Uzbekistan, Turkmenistan, the United States, and Africa) showed variations in Tashkent and California environments (Table 2).

**Table 2:** Comparison of fiber traits among various geographical groups of *G*. *barbadense* cultivars evaluated in the Uzbekistan and USA environments.

Germplasm origin	Micronaire		Strength		Length		Uniformity	
	Uzb	USA	Uzb	USA	Uzb	USA	Uzb	USA
**Africa**	Ẋ = 4.26 S = 0.59	Ẋ = 4.1 S = 0.44	Ẋ = 37.85 S = 3.77	Ẋ = 36.01 S = 3.21	Ẋ = 1.29 S = 0.10	Ẋ = 1.35 S = 0.11	Ẋ = 84.30 S = 1.91	Ẋ = 87.1 S = 1.72
**Uzbekistan**	Ẋ = 4.31 S = 0.49	Ẋ = 4.1 S = 0.49	Ẋ = 39.08 S = 2.44	Ẋ = 36.78 S = 2.09	Ẋ = 1.31 S = 0.08	Ẋ = 1.36 S = 0.07	Ẋ = 84.89 S = 1.52	Ẋ = 87.3 S = 1.17
**USA**	Ẋ = 4.38 S = 0.64	Ẋ = 4.0 S = 0.44	Ẋ = 38.39 S = 3.09	Ẋ = 36.83 S = 2.09	Ẋ = 1.28 S = 0.07	Ẋ = 1.35 S = 0.07	Ẋ = 83.66 S = 2.13	Ẋ = 87.2 S = 0.80
**Turkmenistan**	Ẋ = 4.18 S = 0.51	Ẋ = 4.0 S = 0.47	Ẋ = 38.57 S = 3.11	Ẋ = 36.32 S = 3.01	Ẋ = 1.31 S = 0.09	Ẋ = 1.36 S = 0.09	Ẋ = 84.77 S = 1.57	Ẋ = 87.3 S = 1.61

Note:Ẋ—mean; S—variance. A number of accessions for Uzbekistan (247) and the USA (278) environments were as specified in Table 1.

### Fiber trait correlations

The correlation analyses of the fiber traits of *G*. *barbadense* germplasm and cultivars in Uzbekistan and California environments (Table 3) showed the presence of significant positive and negative relationships between the traits studied.

**Table 3:** Correlations between fiber traits of *G*. *barbadense* cultivars in the Tashkent and California environments.

**Fiber traits**	**FM**	**FS**	**FL**
**Tashkent, Uzbekistan**			
**FM**	1		
**FS**	-0.258**	1	
**FL**	-0.560**	0.505**	1
**FU**	-0.297**	0.634**	0.610**
**California, USA**			
**FM**	1		
**FS**	-0.068	1	
**FL**	-0.427 **	0.155*	1
**FU**	-0.241 **	0.219**	0.774**

* Correlation is significant at a value *r* ≤ 0.05;

** Correlation is significant at a value *r* ≤ 0.001; FM—fiber micronaire, FS—fiber strength, FL—fiber length, FU—fiber uniformity

Positive correlation was observed between FL and FU, FL and FS, and FU and FS. The negative correlations were observed between FM and FS (not significant in California), FM and FL, FM and FU in both environments. Significant trait correlations were observed between the same fiber traits as well as among different fiber traits in Tashkent and California-grown accessions (Table 4).

**Table 4:** Comparative analysis of fiber traits correlation depending on the growing conditions.

**Environment_Fiber triats**	**US_FM**	**US_FS**	**US_FL**	**US_FU**
**UZB_FM**	0.603(**)	-0.144(*)	-0.491(**)	-0.374(**)
**UZB_FS**	-0.096	0.520(**)	0.278(**)	0.280(**)
**UZB_FL**	-0.372(**)	0.249(**)	0.689(**)	0.548(**)
**UZB_FU**	-0.170(**)	0.267(**)	0.324(**)	0.339(**)

* Correlation is significant at p = 0.05;

** Correlation is significant at p = 0.001

These trait-correlations revealed the importance of the environment influencing fiber development. Similar results or similar pattern of variability for fiber traits were observed based on the analysis of variance (ANOVA), in which environmental growth condition impacted the fiber trait differences. ANOVA also revealed that the differences between these groups (Uzbekistan and California) strictly depended on fiber traits’ growing conditions (Table 5).

**Table 5:** ANOVA^1^ results of fiber traits depending on the growth in Uzbekistan (Tashkent) and the USA (California).

Statistical analyses	FM	FS	FL	FU
χ2; *p-value*	18.84; 0.000014	97.10; 0.000000	57.62; 0.000000	244.92; 0.000000
F-Ratio; *p-value*	23.15; 0.000002*	94.15; 0.000000*	57.88; 0.000000*	390.55; 0.000000*
Power (α = 0,05)	0.9977	1	1	1
SS country	5.61	724.10	0.397	892.66
SS S(A)	131.51	4176.40	3.726	1241.13
MS country	5.61	724.10	0.397	892.66
MS S(A)	0.24	7.69	6.86E-03	2.28
SS Total	137.12	4900.51	4.123	2133.79
DF country	1	1	1	1
DF S(A)	574	574	574	574

^1^to determine the statistical significance the F-value used for reliable level of α = 0.05.

*values are significant at α = 0.05

Growing conditions have a direct impact on the performance of the fiber. In Uzbekistan, all accessions showed low values of FL and FU, and high values of FM and FS. Thus, in California, traits were observed to be most favorable for FL, FU, and FM in *G*. *barbadense* germplasm studied. Several accessions with strong stability for a single trait and/or all traits in the Tashkent and California environments were identified (Table 6).

**Table 6:** Samples having a strong stability in the Uzbekistan and USA.

Traits	Number of cultivars	Value
Fiber strength	92	> 37g/tex
Fiber micronaire	41	≥ 3.7≤4.2
Fiber length	9	≥ 1.5 inches
Fiber uniformity	7	> 87%
Combination of all traits	7	

### Marker analysis

From the 750 SSR primer pairs, 108 (14%) were found to be polymorphic among *G*. *barbadense* germplasm and cultivars. Identified 108 SSRs primer pairs amplified 301 marker loci in our *G*. *barbadense* panel (S2 Data). The number of alleles ranged from 2 to 5 with an average number of 2.78 allele per SSR. Sixty SSRs (55%) amplified three or more alleles. The majority of SSRs (81%) were represented by two or three alleles on these accessions (Table 7). The average polymorphic information content (PIC) among the markers was 0.29 (SD = 0.16). Mean heterozygosity (H) for all markers among the 288 accessions of *G*. *barbadense* was 0.33 (SD = 0.2), with the minimum and maximum values of 0.02 and 0.71, respectively. Of the 108 markers, in 49 (45.4%) markers, the heterozygosity values ranged from 0.02–0.25 and in 59 (54.6%) markers, the values ranged from 0.25–1.0.

**Table 7:** A distribution of alleles among the 108 SSR markers.

Number of SSRs	Alleles	Total Alleles
48	2	96
40	3	120
15	4	60
5	5	25
**Total: 108**		**301**

Comparative analysis of heterozygosity and PIC values showed higher values in heterozygosity over PIC in each of the markers with an average increase of about 10.1%. Analysis of the distribution of frequencies of polymorphic alleles showed that the average frequency was 0.424 (SD = 0.33) with minimum and maximum values of 0.02 and 0.996, respectively. Out of 301 amplified/identified marker-alleles, 189 loci had a higher allele frequency than 5% and rest 113 loci turned out to be rare (≤ 5%) and created minor allele frequency (MAF) in the assessed germplasm panel. Minimum, maximum and average values of minor allele frequency were 0.02, 0.05 and 0.028, respectively. Identified rare marker alleles occurred in 138 (~ 48%) accessions. The number of rare alleles on these accessions ranged from 1 to 32.

Based on our analyses, 38 SSR markers revealed high polymorphisms in this panel of long-staple cotton. These SSRs produce enough polymorphisms and can be recommended for molecular analyses of the *G*. *barbadense* genome (S1 Table). The chromosome locations of most SSR markers and their positions on chromosomes were determined by the consensus genetic map of tetraploid cotton reported by Blenda et al. [62].

### Genetic distances and phylogeny of long-staple cotton germplasm

**Fig 1:**
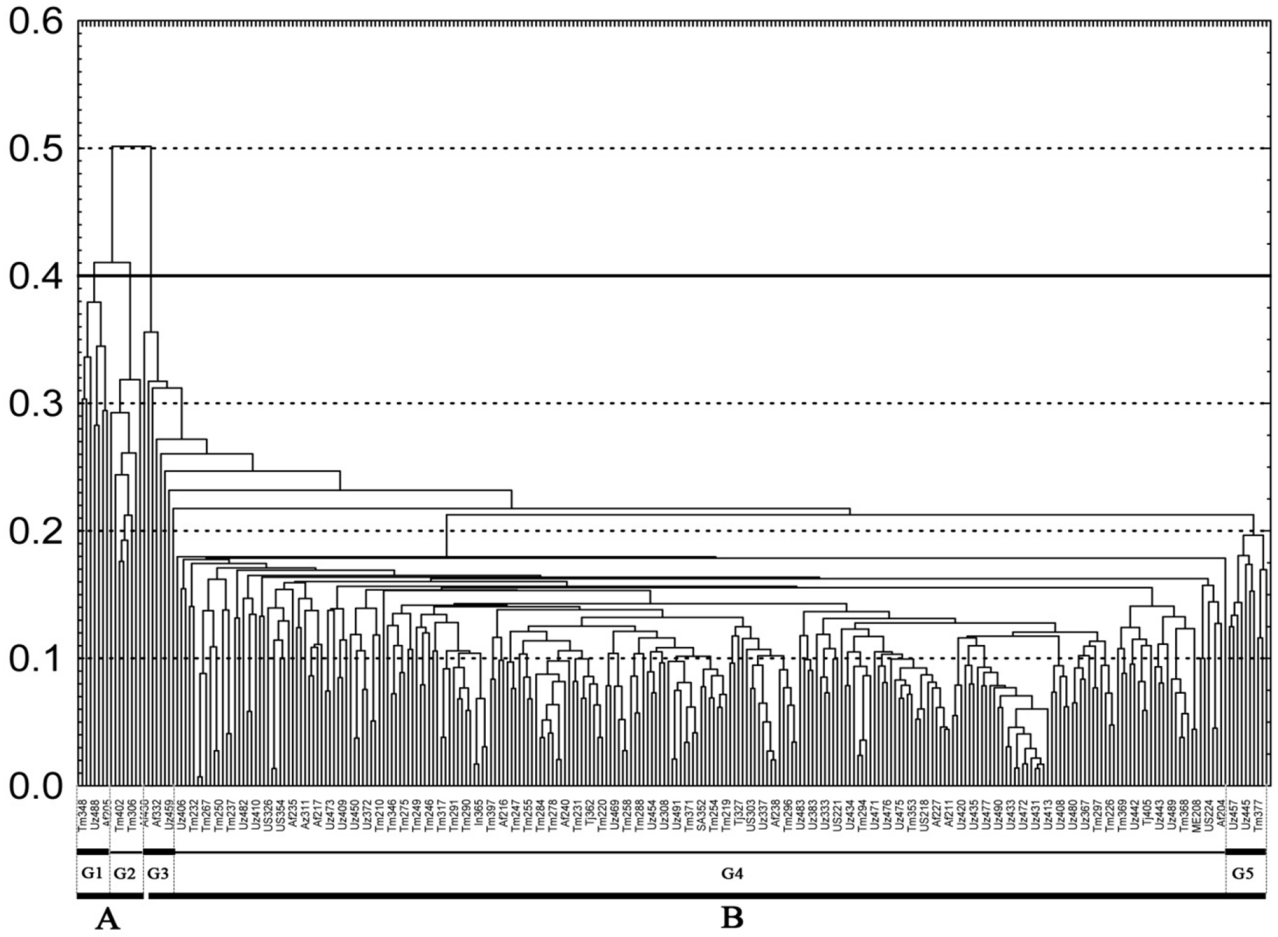
The UPGMA dendrogram of 288 *G*. *barbadense* accessions, constructed using the genotype of 301 polymorphic SSR alleles. Horizontal lines denote thresholds of genetic distances. Groups A and B are obtained on the basis of differences in > 50%, whereas subgroups G1, G2 and G3 obtained based on the upper boundary distinctions in 40%, and the subgroups G5 and G4—the upper bound of 20%.

The average value of genetic distance (GD) among all 288 *G*. *barbadense* accessions was 0.19 with the smallest and largest distances of 0.01 and 0.67, respectively. The developed UPGMA dendrogram revealed two main groups «A» and «B» with the GD threshold of > 50%, and five clearly distinct subgroups (Fig 1). The GD between the groups "A" and «B» was 0.65. The group "A" was genetically diverse, and samples within the group "A" were much different from each other. The average GD between samples in the group "A" was 0.31; for example, the accessions in this group were similar with an average of 69%, representing a wide genetic diversity.

Based on the fact that if GD between local populations of a single species is usually less than 0.05 [63–65], the samples belong to the same population group [66]. While if the distance is greater than 0.05 or 5% the individuals or accessions are likely to belong to different population subgroups. Thus, group “A” was separated. The group "A" was observed to be composed of two sub-groups G1 and G2. The GD between these subgroups was 0.40. The sub-group G1 predominantly consists of accessions of Africa-Egyptian genotypes (Giza, Barakat), and sub-group G2—American genotypes (Pima S1).

The group "B" included 273 cultivars or 94.8% of the analyzed accessions. The group consisted of accessions from different geographical regions of the world. The average GD in this group was much lower than in the group "A". Thus, minimum and maximum GDs in the group "B" were 0.01 and 0.65, respectively, with a mean of 0.18. Group "B" consisted of subgroups G3, G4 and G5. Subgroup G3 includes nine samples (5 African, 3 Uzbekistan, and 1 China). The genetic distance between samples in the subgroup G3 varied from 0.22 to 0.38 with a mean of 0.31. The sub-group G4 is the largest, containing 254 accessions with average GD of 0.18. The subgroup included samples from many geographic regions. The last group G5 is represented by the Central Asian germplasm.

### Molecular diversity and structure of the *G*. *barbadense* panel

**Fig 2:**
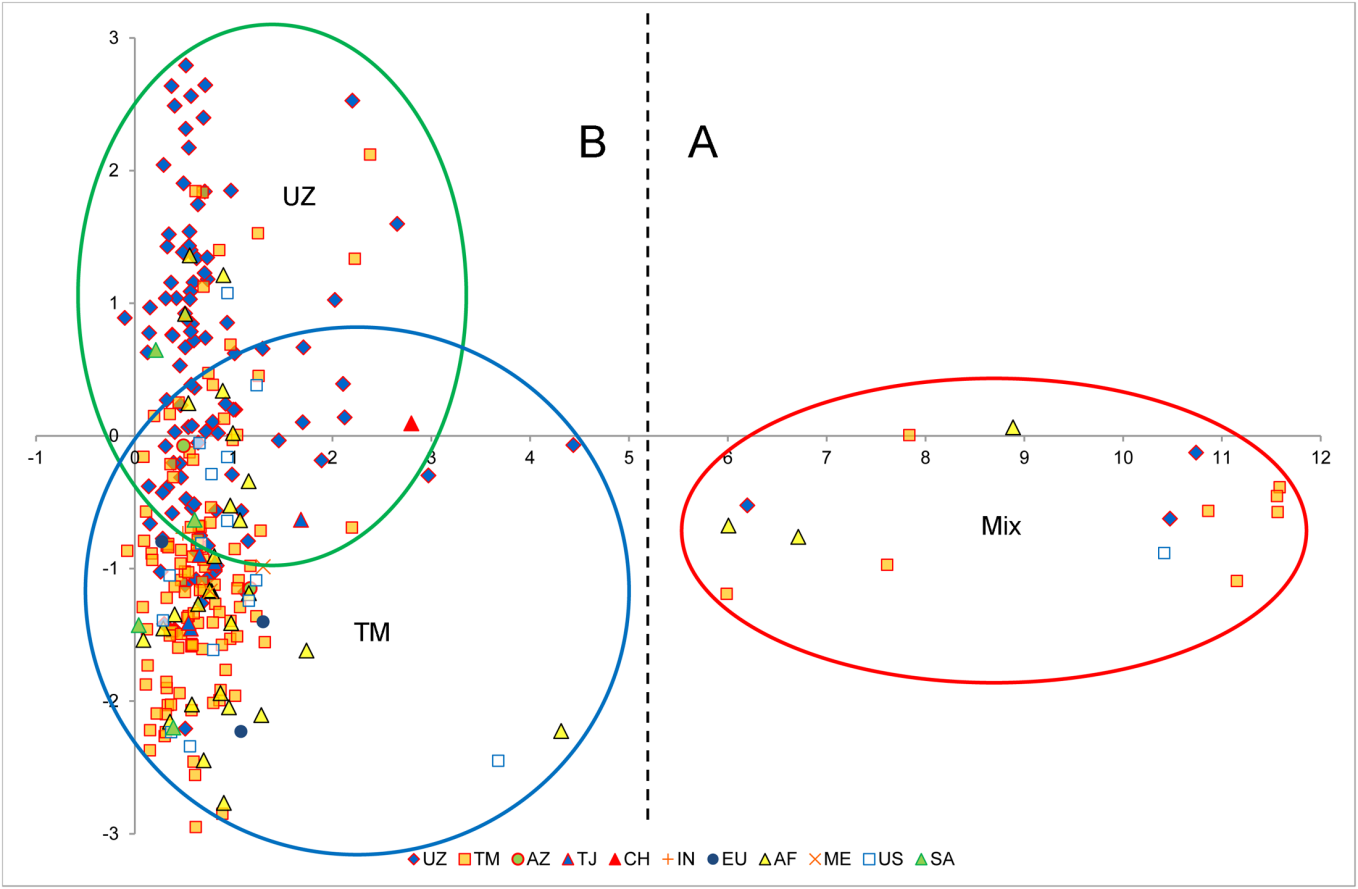
Principal component analysis, of 288 *G*. *barbadense* cultivars in the space of two main coordinate jointly by SSR genotypes. PC—the main components; (A) and (B)—subgroups represented in the majority of varieties of Uzbekistan (UZ) and Turkmenistan (TM), respectively. (Mix)—represented by the most genetically differentiated samples from several geographic regions i.e., from Turkmenistan (8), Africa (3), Uzbekistan (3), and American (1). UZ—Uzbekistan, TM—Turkmenistan, TJ—Tajikistan, AF—Africa, US—US, SA—South America AZ—Azerbaijan and ME—Middle East.

In order to confirm the phylogenetic analysis and to support the population structure analysis, the principal component analysis (PCA) of SSR marker data was performed. PCA reduced the dimensionality of data and displayed all 288 *G*. *barbadense* accessions in a "two-dimensional" space, unlike the phylograms above. In addition, it more clearly reflects the grouping of samples and differences at the genetic level. As a result of PCA, it was determined that the first twelve components explained 51% of the variations. Of them the PC1 explains 15% of the variance, and clearly delineates the population into two subpopulations—large and small (Fig 2). The PC2 causes a 5% dispersion of 273 samples split into two main subpopulations overlapping subgroups, conventionally designated as Group A and Group B (Fig 2 and Table 8). Group A includes 108 accessions, which included most represented accessions from Uzbekistan—81 (75%), and group B comprises 165 accessions, in which the majority of genotypes are accessions from Turkmenistan—99 (60%; Fig 2 and Table 8).

**Table 8:** Differentiation of 288 *G*. *barbadense* accessions based on genetic and principal component analysis.

Germplasm origin	Subpopulation 1		Subpopulation 2
	Subgroup A	Subgroup B	
**Uzbekistan**	81	24	4
**Turkmenistan**	17	99	7
**US**	3	11	1
**Africa**	6	25	3
**other**	1	6	-
**Total:**	**108**	**165**	**15**

### Analysis of Molecular Variation (AMOVA)

To assess the genetic differentiation among and within predefined groups of a whole panel of 288 *G*. *barbadense* accessions, the Wright`s index F_st_ (pair wise) was analyzed using statistical analysis of AMOVA. The genetic differentiation among and within groups were significant (*p* ≤ 0.001), where 67.2% of total genetic variation was attributed to the difference within subpopulations, while the genetic variation between the predefined groups accounted for 32.8% of the total genetic variation (Table 9). Pairwise comparisons of the Fst index between the three groups revealed that the greatest genetic differentiation was present between the African group and the Turkmen group (F_st_ = 0.58; *p* < 0.001), and a little less variation, between the African and Uzbek group (F_st_ = 0.57; *p* < 0.001) (Table 10). Low-moderate genetic differentiation was found between the Uzbek and Turkmen subpopulation (F_st_ = 0.117).

**Table 9:** The AMOVA results.

Source of variation	df	Sum of squares	Variance Components	Percentage of variation	p-value
**Among populations**	2	1312.320	11.769	32.797	≤0.001
**Within populations**	285	6791.922	24.115	67.203	≤0.001
**Total**	287	8104.242	35.884		

**Table 10:** Pair-wise comparisons of F_st_ values specific to each ecotype.

**Origin**	**Africa**	**Uzbekistan**	**Turkmenistan**
**Africa**	0.00000		
**Uzbekistan**	0.57568	0.00000	
**Turkmenistan**	0.58432	0.11723	0.00000

### Population structure and kinship

**Fig 3:**
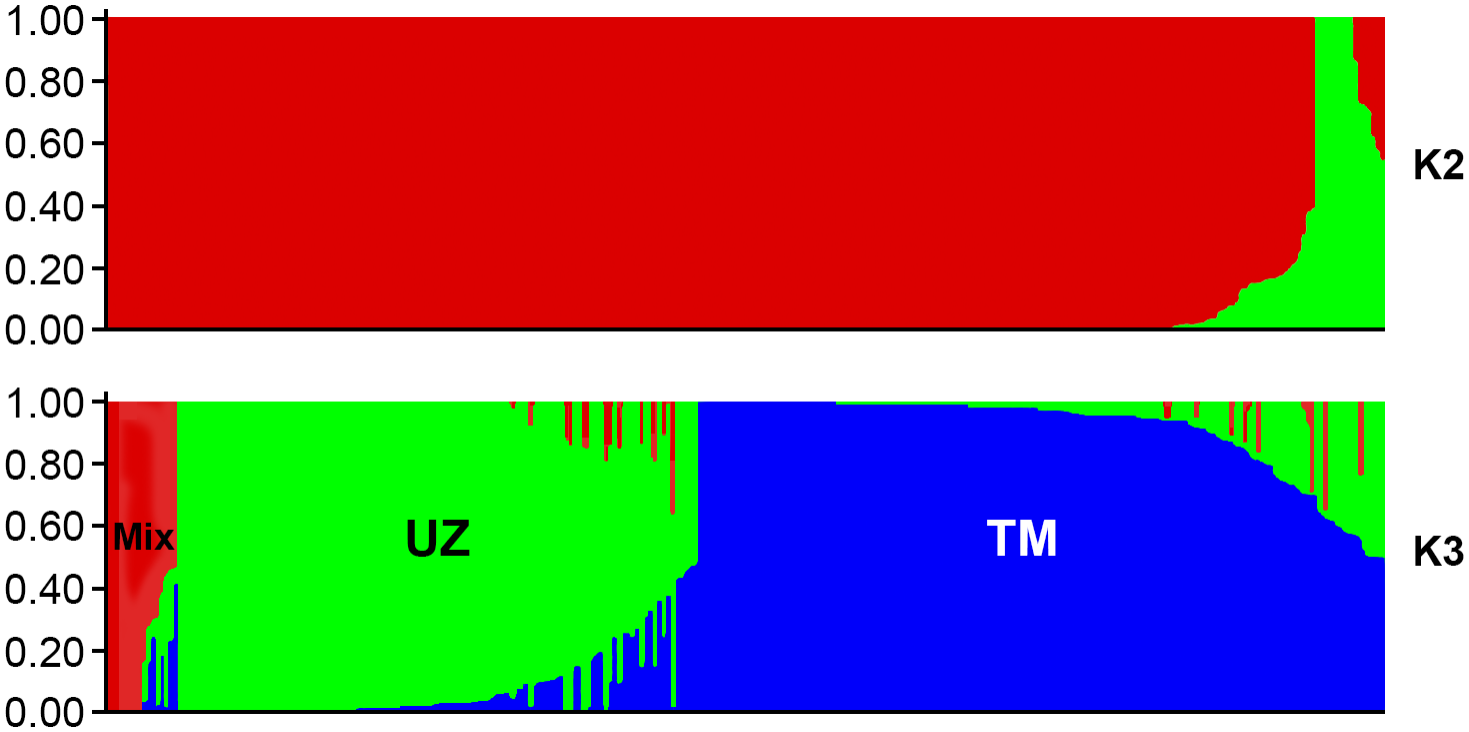
Summary plots of Q-matrix for the *G*. *barbadense* germplasm inferred from STRUCTURE analysis. K2—the division into two subpopulations:a small (green) and large (red). K3—further expansion of subpopulations on ecotypes (consistent with the results shown in Fig 2). Mix- represented by the most genetically differentiated samples from several geographic regions. UZ—Uzbek, and TK—Turkmen cotton accessions.

The model-based approach revealed the presence of at least two main subpopulations (Fig 3, K2). These two subpopulations share accessions of the total panel, 5.2% and 94.8%, respectively. Further expansion of the total population allowed us to divide it into three subpopulations, where a small mixed cluster remained unchanged (5.2%), and a large cluster was divided into two sub-populations sharing 37.5% and 57.3% of accessions, respectively (Fig 3, K3). The results of the structured population of *G*. *barbadense* 288 accessions were consistent with the phylogenetic analysis. Accessions, based on the genetic profile, clearly were divided into samples of mixed (accessions from all regions), Uzbek (UZ) and Turkmen (TM) cotton accessions.

The pairwise kinship analysis revealed that the majority of the pairs of cotton accessions (56%) had zero kinship values, whereas 22–23% of the pairs had a pairwise kinship value of 0.01–0.05 and 10% of the accession pairs had 10–20% relatedness. Only about 1.3% accessions had a pairwise kinship value of ≥25%.

### Linkage disequilibrium (LD) and LD decay

The SSR data with 5% MAF removed set of 189 alleles were used to evaluate the extent of LD at genome level that detected pairwise LD in 17766 locus pairs in the *G*. *barbadense* panel. At significant threshold values (r^2^ ≥0.05 and p ≤0.005), 16.8% (4576) of SSR marker pairs showed significant LD. By increasing the threshold to substantially higher values, r^2^ ≥0.1 (p <0.001) and r^2^≥0.2 (p <0.0001), LD was maintained in 2188 (8%) and 1187 (4.3%) of pairwise combinations of SSR markers, respectively. The linear plot of triangular graph of pairwise genome-wide LD between markers revealed significant LD blocks. This information is necessary to calculate to support the association mapping with the average distance of LD decay.

**Fig 4:**
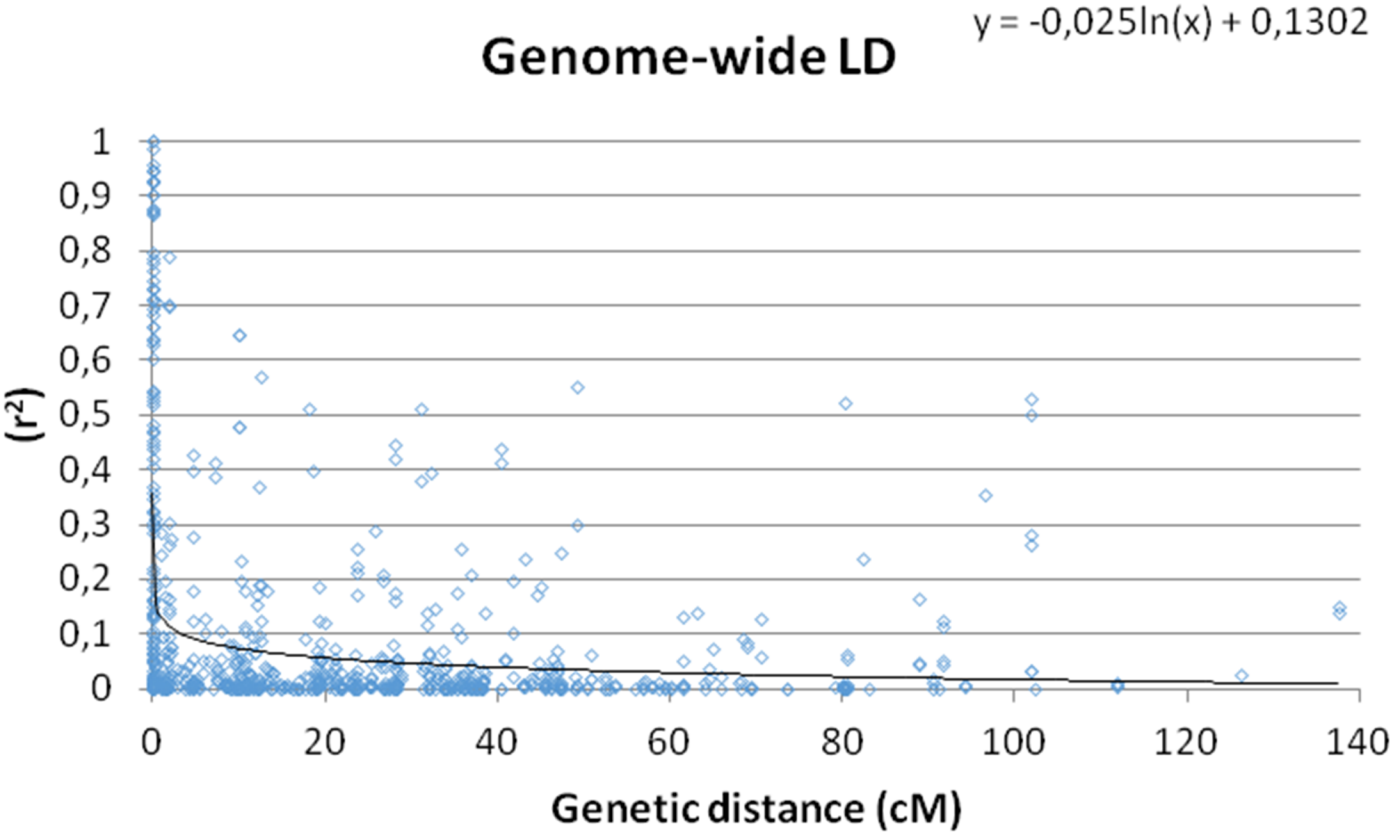
Scatter plot of significant r^2^ values and genetic distance (cM) (p<0.001) of locus pairs on whole genome of *G*. *barbadense* germplasm.

To reveal LD decay in *G*. *barbadense* genome, LD decay scatter plots of r^2^ vs. genetic distance (cM) was generated to estimate LD decay using curvilinear regression (Fig 4). Results revealed that LD decay in *G*. *barbadense* genome was on average of 24.8 cM at the threshold of r^2^ ≥0.05. The genome-wide LD decay (r^2^ ≥0.1) was 3.36 cM in *G*. *barbadense* (Fig 4). These findings suggested the possibility of performing an efficient LD-based association mapping in the germplasm accessions of *G*. *barbadense* presented here.

### Association mapping (AM) of fiber traits

**Fig 5:**
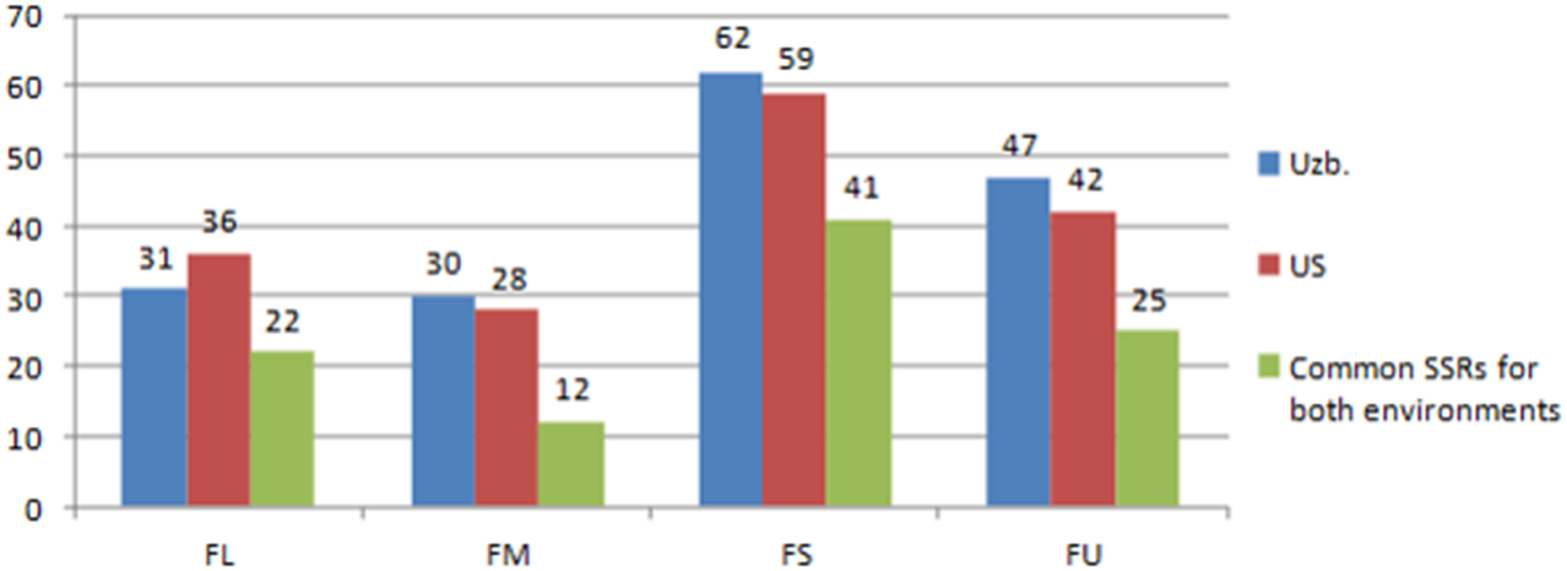
Result of association mapping of fiber quality traits in a particular region. Markers showed signisicant association (MLM; *p* ≤0.05) both in Uzbekistan (Uzb.), and the United States (US) environments.: FL-fiber length, FM- micronaire, FS- fiber strength, FU- uniformity.

AM analysis of SSR loci with fiber quality traits of 247 and 278 *G*. *barbadense* accessions, grown and evaluated in the two different ecological and geographical environmental conditions, Tashkent (Uzbekistan) and California (USA), respectively, was performed using TASSEL software. According to the results, fiber traits varied from 1.6 to 11.4% in the USA and from 1.9 to 12.2% in the Uzbekistan environments. Therefore, not all markers associated with fiber traits in a single eco-geographic region showed association in both environments. However, a set of 100 markers retained a strong correlation and was significantly associated (MLM; *p* ≤0.05) in both environments (Tashkent and California). For example, for fiber length, 22 markers showed significant associations: to 12—with micronaire, 41 and 25 with the strength and uniformity, respectively (Table 11 and Fig 5). Moreover, at critical values of minimum BF≤0.13 11 SSRs retained strong associations with fiber traits (Table 11), of them 3 markers with FM (BNL3441_225, BNL3601_175, NAU2913_250), 2 with FS (BNL4003_150 and GH39_125), 4 with FL (BNL3599_200, GH75_130, NAU2913_275, NAU2913_250) and 2 with FU (BNL3902_200 andBNL3601_175).

**Table 11:** Summary of SSR markers showed significant association with each of the trait studied in the Uzbekistan (UZB) and USA environments.

Traits	Number of markers with significant associations*			Markers associated with traits at BF ≤0.13 ^1^		
	UZB	USA	Common markers**	UZB	USA	Common markers**
Micronaire	30 (3)	28 (3)	12 (3)	9	6 (1)	3
Strength	62 (9)	59 (6)	41 (5)	6	3 (1)	2
Length	31(6)	36 (7)	22(6)	10 (1)	2	4
Uniformity	47(0)	42 (1)	25	8	5	2

Note:

* The table shows the markers that showed a significant association (p ≤0.05) on the basis of MLM analysis 1.000 times permutation. In parentheses is the number of matched SSR markers associated with the described fiber traits, which are reported in other studies;

**the markers showed similar associations in both conditions.—Evaluation of these parameters in Uzbekistan and the United States have not performed.

^1^BF—Bayes factor (likelihood ratio), where the value of BF≤0.13 correspond to strong (z = 2.17) evidence against the null hypothesis (Ho) [59, 65, 66].

When all fiber trait-associated SSR markers from our study were compared to reported SSR markers in previously published QTL-mapping studies, 14 SSRs [19, 67–76] revealed the same trait associations identified in our study (Tables 11 and 12). The remaining 86 SSR markers were identified for the first time in this study (Table 12).

**Table 12:** SSR markers showed significant fiber trait-association (MLM; p≤0, 05) in both environments (USA and Uzbekistan).

#	SSR marker*	USA		Uzbekistan		Match **	Chromosome***	Association with other traits (reference)
		*F* (MLM)	*p* (MLM)	*F* (MLM)	*p* (MLM)			
**Fiber length**								
1	BNL1421_220	5,3046	0,0221	4,5953	0,0331	[71]	13; 18	FL, FS, FE [77]
2	BNL1421_250	8,7632	0,0034	10,3148	0,0015	[71]	13; 18	FL, FS, FE [77]
3	BNL1495_150	7,4088	0,0070	11,1068	0,0010	[75]	13; 18	FE [78]; FL, FS, FE [77]; Lint% [72]
4	BNL1521_170	20,2535	0,0000	17,2683	0,0000	[19]	24; 15	FS [19,79]; FM, FE [70]; FM [21]
5	BNL1611_190	11,1161	0,0010	7,0728	0,0084	-	19	Lint index
6	BNL1705_200	13,6747	0,0003	8,1739	0,0046	[76]	21	FF; FE [76]; Lint% [80]
7	BNL3171_250	11,2913	0,0009	10,7198	0,0012	[81]	14; 21	FS, FL [81]; NSPB [82]
8	BNL3398_190	13,7112	0,0003	7,8360	0,0055	-	3; 13	
9	BNL3442_130	7,3967	0,0070	5,1340	0,0244	-	11	FS [81]
10	BNL3599_200^1^	14,5256	0,0002	16,0080	0,0001	-	12; 25; 26	Near (2cM) the CF44ss gene, differential expressed during fiber development [83]
11	BNL3599_225	14,0670	0,0002	12,6243	0,0005	-	12; 25; 26	
12	BNL3902_200	10,5747	0,0013	4,0639	0,0450	-	15	FM, FF [70,19]; FE, FS [78]
13	BNL625_260	7,5997	0,0063	9,1459	0,0028	-	11	
14	GH118_150	14,4985	0,0002	13,6357	0,0003		9	
15	GH39_100	8,4011	0,0041	5,9578	0,0154	[75]	8	
16	GH58_150	11,2451	0,0009	7,3450	0,0072	-	9	
17	GH75_130^2^	3,9857	0,0470	7,2289	0,0000	-	1	
18	GH77_130	15,6853	0,0001	12,1496	0,0006	-	25	
19	GH83_150	14,4985	0,0002	13,6357	0,0003	-	5	
20	NAU2913_275^2^	28,0884	0,0000	33,1437	0,0000	-	26	
21	NAU2913_250^1^	10,9679	0,0011	7,3916	0,0072	-	26	
22	TMB1660_200	4,7863	0,03	4,0461	0,0459	-	13	
**Micronaire**								
23	BNL1317_200	5,3110	0,0221	4,4295	0,0364	[,^19^,^2^, ^70^]	9; 23	FL; FS; FF; FM; FE [70,19,84–87]; [21,86,88,89]; [19,84,85,88]; [70]; Lint% [72]; Phe, Val, Ile [83]
24	BNL1611_250	4,6319	0,0324	4,3260	0,0386	-	19	Lint index
25	BNL2655_170	4,5173	0,0346	7,1258	0,0081	[89]	24	-
26	BNL2655_180	6,5472	0,0111	7,2491	0,0076	[89]	24	-
27	BNL3171_270	4,7169	0,0309	5,5791	0,0190	-	21	FS, FL [81]; NSPB [82]
28	BNL3441_225^1^	4,4178	0,0366	5,5588	0,0192	[78]	3	Seed Yield, Lint Yield [76]
29	BNL3599_200	4,569	0,0338	4,6125	0,0331	-	12; 25; 26	-
30	BNL3601_150	7,1592	0,0081	4,3647	0,0382	-	22	-
31	BNL3601_175^2^	14,7358	1,69E-04	7,7107	0,0001	-	22	Maturity, Wall thickness [72]
32	BNL3792_275	3,7064	0,0558	3,9364	0,0489		8	-
33	BNL3977_200	7,3660	0,0071	6,4973	0,0114	-	19	-
34	NAU2913_250^1^	6,8974	0,0092	22,7003	0,0000	-	26	-
**Fiber strength**								
35	BNL1521_170	4,4852	0,0352	11,4486	0,0008	[19]	24	FS [79]; FM [21]; FM, FE [70]
36	BNL1667_275	6,3563	0,0124	10,2118	0,0016	-	15	-
37	BNL2590_300	9,5001	0,0023	12,4752	0,0005	-	9; 23	-
38	BNL2632_250	5,9613	0,0154	9,6192	0,0022	-	11; 21	-
39	BNL2960_170	5,0139	0,0261	5,6441	0,0183	-	10	FL, FE [75]
40	BNL2967_180	4,5632	0,034	10,5314	0,0014	-	12	-
41	BNL3071_150	3,9221	0,0488	6,6112	0,0108		20; 10	-
42	BNL3259_210	6,9678	0,0089	10,0291	0,0017	[81]	3; 14	-
43	BNL3408_150	7,5481	0,0065	6,9507	0,0089	-	3	-
44	BNL3442_130	9,8947	0,0019	9,0888	0,0029	-	11	-
45	BNL3452_180	7,1697	0,0079	8,7972	0,0033	[81]	3; 11	-
46	BNL3479_240	11,6504	0,0008	7,4832	0,0067	-	13; 18	-
47	BNL3482_130	7,7441	0,0058	4,4109	0,0368	[72]	26; 20	ELO [33]; MIC, BW, seed index [72]
48	BNL3590_225	6,3393	0,0125	10,0275	0,0017	-	2; 17	Lint%, BN [80]
49	BNL3599_200	4,9390	0,0272	11,2787	0,0009	-	12; 26	-
50	BNL3955_175	7,1704	0,0079	15,7115	0,0001	-	6; 17; 22	-
51	BNL3955_200	5,3898	0,0211	4,7147	0,0309	-	6; 17; 22	-
52	BNL3992_170	11,6470	0,0008	14,6615	0,0002	-	5	-
53	BNL3994_120	14,2212	0,0002	4,7149	0,0309	-	4; 22; 26	-
54	BNL3994_130	5,5912	0,0189	4,4562	0,0359	-	4; 22; 26	-
55	BNL4003_150^1^	5,3124	0,0221	10,2666	0,0015	-	17	-
56	BNL4061_200	12,8342	0,0004	7,2074	0,0078	-	13	-
57	BNL625_260	18,7211	0,0000	18,2298	0,0000	-	11	-
58	CM209_120	9,1795	0,0027	6,4197	0,0119	-	9	Node number, resistance to *V*. *dahlia* [90]
59	CM209_150	4,9767	0,0266	4,5798	0,0334	-	-	-
60	GH107_250	4,8334	0,0289	5,2264	0,0231	-	4	-
61	GH110_140	9,1454	0,0028	7,4042	0,0070	-	20	-
62	GH117_160	11,2309	0,0009	18,7498	0,0000	-	4	-
63	GH117_170	12,2549	0,0006	15,2407	0,0001	-	4	-
64	GH118_150	20,3746	0,0000	24,2525	0,0000	-	9	-
65	GH171_250	9,2628	0,0026	9,7270	0,0020	-	24	-
66	GH200_150	7,6600	0,0061	19,2938	0,0000	-	22	-
67	GH39_100	17,4070	0,0000	17,7736	0,0000	-	6	-
68	GH39_125^1^	14,8501	0,0002	5,5328	0,0195	-	6	-
69	GH58_150	18,7026	0,0000	24,3992	0,0000	-	10	-
70	GH75_150	7,9448	0,0052	11,8654	0,0007	-	1	-
71	GH77_130	14,0671	0,0002	15,6514	0,0001	-	-	-
72	GH82_190	14,8755	0,0001	20,6424	0,0000	-	12; 6	-
73	GH83_150	20,3746	0,0000	24,2525	0,0000	-	5; 21	-
74	NAU2709_150	19,9144	0,0000	23,9373	0,0000	-	23	-
75	NAU3620_150	11,3272	0,0009	10,2668	0,0015	-		-
**Fiber uniformity**								
76	BNL1495_135	8,0936	0,0048	4,6559	0,0320	-	13	FE [78]; FL, FS, FE [77]; FY [72]; FL [75]
77	BNL1521_170	16,9956	0,0001	17,4674	0,0000	-	24	-
78	BNL1611_190	12,8852	0,0004	17,3193	0,0000	-	19	-
79	BNL2590_300	3,9382	0,0484	6,2594	0,0131	-	9	-
80	BNL2960_170	6,4625	0,0117	5,3490	0,0216	-	10	FL, FE [75]
81	BNL2967_200	7,8402	0,0055	5,8171	0,0166	-	12	-
82	BNL3398_190	10,0170	0,0018	17,7981	0,0000	-	3	-
83	BNL3599_200	18,9071	0,0000	15,2568	0,0001	-	12	-
84	BNL3599_225	16,0116	0,0001	9,1933	0,0027	-	12	-
85	BNL3601_175^1^	12,2255	0,0006	4,6528	0,0320	-	22	-
86	BNL3902_200^1^	7,7856	0,0057	13,9393	0,0002	-	15	-
87	BNL3955_175	9,0779	0,0029	16,1893	0,0001	-	6; 17; 22	-
88	BNL4061_200	6,9722	0,0088	3,9938	0,0468	-	13	-
89	BNL625_260	6,4257	0,0119	8,5918	0,0037	-	11	-
90	GH107_250	4,1634	0,0424	7,9804	0,0051	-	4	-
91	GH118_150	14,3306	0,0002	16,9257	0,0001	-	9	-
92	GH39_125	7,6613	0,0061	9,8348	0,0019	-	6	-
93	GH58_150	14,4281	0,0002	14,8799	0,0001	-	10; 9	-
94	GH77_130	14,1736	0,0002	11,3857	0,0009	-	-	-
95	GH77_175	5,0152	0,0261	8,9700	0,0030	-	-	-
96	GH82_190	14,4588	0,0002	21,1300	0,0000	-	6	-
97	GH83_150	14,3306	0,0002	16,9257	0,0001	-	4; 21	-
98	NAU3620_150	11,5049	0,0008	8,8581	0,0032	-	5	-
99	NAU3860_250	4,2711	0,0399	3,9286	0,0487	-	12; 26	-
100	NAU5015_180	10,4259	0,0014	4,7976	0,0295	-	5	-

*—SSRs with significant (≤0,05) fiber trait associations;

**—The same marker-trait association matched with other studies (reference),

***—Chromosome location of SSR marker;

^1^—Marker showed significant association at Bayes factor BF≤0,15 [61],

^2^—Marker with very strong association at BF ≤0,02; FL—fiber length; FS—fiber strength; FE—fiber elongation; FM—fiber micronaire; Lint%—lint yield, Phe -phenylalanine, Val—valine, Ile- isoleucine, NSPB—number seeds per boll.

## Discussion

Gathering information about genetic diversity and population structure is essential for providing insights into the breeding history and genetic relationship of crop germplasm. In this research, the first SSR marker-based molecular genetic study was conducted of *G*. *barbadense* cotton germplasm from the Uzbekistan cotton collection. It is believed that 10–30% of accessions may be enough to represent 70–90% of the genetic diversity of a whole germplasm collection [91]. The 288 accessions studied here represent almost 29% of the entire long-staple cotton genetic collection preserved in IG&PEB, Tashkent, Uzbekistan.

Assessment of *G*. *barbadense* accessions revealed a wide range of diversity in fiber quality traits within specific environments and between environments indicating the existence of useful genetic variation for these traits within the collection. Correlations of fiber quality traits between the USA (California) and Uzbekistan (Tashkent) environments demonstrated different performance of the same long-staple cotton accessions, which reflects the effect of the environment on the development of fiber quality traits. This should be taken into account when breeding for these traits. Nevertheless, several accessions were identified with stable fiber trait performance in both environments (Table 6). By definition, stability is the ability of an accession or genotype to show minimum variability in the interaction with the environment [92]. Thus, identified stable *G*. *barbadense* accessions that demonstrated the best values for single or all fiber traits in both (USA and Uzbekistan) environments should be primarily considered for breeding programs.

Genetic diversity analysis revealed a narrower genetic base of long-staple cotton germplasm based on SSR markers compared to Upland (*G*. *hirsutum*) cotton. This result is consistent with earlier studies [9, 33, 19, 93–95]. In addition, the genetic diversity was observed to be lower than previous reports from other studies of *G*. *barbadense* germplasm [95–99]. An explanation for this finding could be that previous studies used small sample size and/or low numbers of markers. Another explanation of this phenomenon is that in this study the majority of *G*. *barbadense* accessions from the IG&PEB Uzbek germplasm collection belong to Uzbekistan and Turkmenistan cotton germplasm that are closely related genetically and historically [5].

In this context, comparison of accessions for all clusters showed that the Turkmen cultivars have wider introgression/selection compared to the Uzbek accessions. The presence of groups (clusters) is a reflection of the genetic differentiation of populations as a result of the introduction of genes of genetically distinct forms. Thus, according to the cluster analysis, it can be hypothesized that studied *G*. *barbadense* collection was formed by the introduction/introgression of African (including Egyptian), African-American and American genotypes. In addition, the genetic relationship, identified between each of the studied accessions, is important for selecting breeding material and in the creation of improved germplasm and cultivars. It is also important to notice that as a result of many years of breeding, the population of *G*. *barbadense* cultivars formed genotypes specific to agro-ecological conditions of the Central Asian region, and clearly was traced by the genetic isolation of the Uzbek and Turkmen cultivars.

The average number of alleles per SSR marker (2.78) was higher than reported elsewhere (1.72—[97]; 1,66—[98]; 1.60—[95]), and the average PIC value (0.29) was lower than the previously reported from the Chinese’s *G*. *barbadense* germplasm (0.32—[98], but close to values reported for *G*. *hirsutum* germplasm (0.28 [18] and 0.30 [80]). On the other hand, the same SSR marker set showed different fragment sizes and polymorphism compared to the *G*. *hirsutum* accessions from the Uzbek germplasm collection, in which the average allele number was higher (5.5) per SSR, whereas PIC value was much lower– 0.082 [33]. As a result, the selected 108 markers, used in this study, are highly suitable to detect allelic variation in *G*. *barbadense* germplasm.

### Population structure and differentiation of *G*. *barbadense* germplasm

To avoid spurious associations in LD-based AM, a detailed knowledge about population structure in a germplasm panel is of great importance. A model-based MLM approach using population structure information [46] is the most reliable method to correct spurious associations. However, under certain scenarios it is difficult to obtain accurate estimates of the actual number of subpopulations (K) [47, 93]. Generally, K is assumed to be the value with the highest estimated LnP(D) generated by STRUCTURE [46]. The LnP(D) value in real data tends to increase with increasing K and might not show a mode for the true K. Therefore, to avoid false associations an *ad hoc* measure ΔK proposed by the Evanno et al. [38] approach was used to detect the true K present in the SSR marker data.

The *ad hoc* measure ΔK [47] values proposed in this study indicated two groups as the most biologically meaningful population structure of the 288 *G*. *barbadense* germplasm panel. Similar clustering results have been reported for population structure of long-staple germplasm from other studies of *G*. *barbadense* [95, 97], including a recent comparative study of genome-wide divergence and population demographic histories for *G*. *hirsutum* and *G*. *barbadense* using genome-anchored SNPs [100]. In this study, several different methods (UPGMA clustering, PCoA, and Bayesian-based approach) were used to determine the level and pattern of genetic diversity and population structure present in the *G*. *barbadense* germplasm accessions based on SSR markers. Thus, grouping based on clustering analysis was an agreement with available background information of these accessions. As a result, the methods adopted here roughly reveal a similar level of population structure.

The AMOVA revealed a clear genetic structure of the germplasm accessions. The high variability of genetic loci within a population might be due to several factors. For example, widespread species pollinated by insects have high intrapopulation variability [101]. The high degree of cross-pollination patterns within the population serve as indicator of intensive breeding events such as hybridization. On the other hand, from an evolutionary point of view, domestication of long-staple cotton has been relatively recent (~ 2500 years BC) [102, 103]. It is known that a population that has passed through a bottleneck has a temporarily disrupted mutation balance among the loci with an excess of heterozygosity [104]. The results of this study re-highlight the presence of the bottleneck in the recent past of cotton domestication.

The introduction of cotton germplasm and cultivars to new environments leads to formation of novel allele combinations in different loci allowing their adaptability to local stresses. This gives rise to several breeds within a gene pool. One example of this ‘cultivar-introduction’ is the "Acala" cultivar from the USA, which was introduced in Central Asia [105]. Analysis of 56 *G*. *barbadense* cultivars from China revealed 8% of genetic differentiation among populations (probably because a common cultivar-introduction) and 92% within populations [98]. In our study, the level of inter-population differentiation was much higher and accounted for 32.8%, while within population variation was 67.2%. This was similar to results of Upland cotton germplasm collection analysis where the genetic differentiation within and among populations of *G*. *hirsutum* accessions was 31.4% and 65.84%, respectively [106].

The AMOVA results demonstrate a significant correlation between genetic differentiation of accessions and their geographic origin. In different populations of the same species, there is always present historical evidence of interbreeding, even if an admixture does not exist at present. According to Wright, an F_st_>0.25 corresponds to a high level of genetic differentiation [107,108]. In this study, the F_st_ value in the *G*. *barbadense* diverse germplasm was equal to 0.328 (p≤0.001), indicating distinct population structure. Pairwise F_st_ analysis revealed strong differentiation between African and Turkmenistan (F_st_ = 0.584), and African and Uzbekistan (F_st_ = 0.575) germplasm. The differentiation between Uzbekistan and Turkmenistan gene pools was much lower (F_st_ = 0,117). This could be due to interbreeding events not only within a geographic niche, but also from similar introductions. This result also indicates that both gene pools (Uzbekistan and Turkmenistan) arose from common ancestors with a tendency of slight isolation, according to the ratio of allele frequencies identified in these groups or populations.

According to genetic relationship-patterns (Fig 1), it can be assumed that during initial development of the “group B” germplasm, ancestors from the Egyptian and Egyptian-American or/and American gene pools might be involved. It was also interesting to notice that group "A" was observed to be composed of two sub-groups G1 and G2, consisting of accessions of Africa-Egyptian (Giza, Barakat) and American genotypes (Pima S1) (Fig 1). Even though *G*. *barbadense* is indigenous to the northern part of South America and extends into Mesoamerica and the Caribbean [24], the Egyptian-Giza and America Pima-S series have been reported to have an interconnected breeding history. The Sea Island lineage also known as long-staple cotton contributed to the development of the Egyptian cotton [7, 24, 25]. This was later reintroduced in the 1940s into the United States as a part of the genetic base of the Pima gene pool of Pima-S series germplasm releases by USDA-ARS [22, 25]. This is the first report in which molecular data and historic breeding records provide similar evidence for the *G*. *barbadense* history. It could also be concluded that ancestors of the Egyptian and Egyptian-American or/and American germplasm were used in the development of the Turkmen and Uzbek *G*. *barbadense* germplasm. Thus, our results provide important insights into the evolutionary and breeding processes that influenced the structure of genetic variation within and among populations, which is the key point in association genetics studies.

### Application of LD-based association mapping approaches

Association mapping (AM) is a very effective method of combining information on the genotype, phenotype, population structure and the LD in plants [28, 54]. The estimation of LD decay during AM is of great importance. In this study, the first attempt to apply the LD-based AM of fiber quality traits of *G*. *barbadense* germplasm from the Uzbek cotton collection was made. The most appropriate measure of the LD for AM studies in plants is the squared correlation coefficient r^2^ [26], which also points to marker-trait correlation [26,109–111]. In this study, 16.8% of SSR marker pairs showed significant pairwise linkage disequilibrium at r^2^≥0.05. At the higher values of r^2^≥0.1 and r^2^≥0.2, 8% and 4.3% of SSR marker pairs showed significant LD, respectively. The value of r^2^≥0.1 was a threshold for significant LD [112]. The results differ from those obtained from studies of different *G*. *hirsutum* germplasm collections [33, 80, 113, 114]. This indicates some differences in the formation of the LD pattern between pairs of loci in the genomes of *G*. *hirsutum and G*. *barbadense* species, which requires detailed comparative studies in the future.

The observed percentage of SSR loci in LD for *G*. *barbadense* genome in our study, as mentioned above (4.3–16.8%), is significantly lower than that of other crops such as corn, barley and wheat, where the percentage of markers in LD has been reported at 49–57%, 52–86% and 45–100%, respectively [115–119]. The low level of pairwise LD between SSR loci might be due to high levels of recombination rate in the genomes of allopolyploid cottons [120], as well as, mutations and experimental hybridization in the recent history of cultivated cotton germplasm [33]. In this study, an average genome-wide LD decay for *G*. *barbadense* accessions was 3.36cM at r^2^≥0.1 and 0.6cM at r^2^≥0.2. A recent study of 219 *G*. *barbadense* cultivars and landrace accessions of widespread origin using the genome-wide SNPs suggested a genome-wide LD decay was longer with an average of 128Kb compared to *G*. *hirsutum* with an average decay of 117Kb [100]. The fast LD decay of *G*. *barbadense* germplasm illustrates the significant potential for LD-based association mapping for agronomic traits. Taking into account that the average length of recombination block in the genome of tetraploid cotton is around 5,200 cM, with an average of 400kb per 1cM [121], the block size of ~ 5 cM is sufficient for reliable association mapping [33]. Therefore, our findings suggest a great possibility for association mapping of *G*. *barbadense* genome.

Several studies of the LD decay in a whole genome scale in diverse *G*. *hirsutum* germplasm collections found that the LD decay varied from 25 to 5 cM at *r*^2^ threshold of 0.1 and from 6 to 1 cM at r^2^≥0.2 [9, 18, 19, 33, 113, 122]. This indicates that the size of the LD blocks may vary depending on the sample size and the population studied although the structure of LD haplotype blocks found to be considerably similar between *G*. *hirsutum* and *G*. *barbadense* [100]. Moreover, the composition of germplasm plays a key role in the LD variations, in other words the genetic distance over which LD decays depends on the genetic diversity present in the population [123]. Therefore, further characterization of the population structure and LD levels in *G*. *barbadense* germplasm collected from all over the world will be a benefit for association mapping of complex traits in long-staple cotton. In our study, the average size of the LD blocks in the genome of *G*. *barbadense* is less than that of *G*. *hirsutum*, which suggests a greater genetic variability. A large part of the genetic variability observed in modern *G*. *barbadense* germplasm may be due to introgression with *G*. *hirsutum* [124]. This also may be due to intensive breeding programs of Upland cotton that are ten times more than those dedicated to *G*. *barbadense* accessions [125,126].

### Fiber quality trait associated markers

Linkage mapping is a powerful tool for identifying the genetic basis of quantitative traits in plants. However, association mapping is another effective approach for connecting phenotypes and genotypes in plants when information on population structure and LD is available [54]. The LD-based AM has recently gained popularity among plant geneticists and become a powerful approach to dissecting complex traits in many crops [28, 29]. In the current study, a number of major fiber trait-associated SSR markers were identified in the two diverse environments of Uzbekistan and USA. Only markers that showed significant associations in both GLM and MLM were considered for further analysis. Among them, 100 SSR markers were associated with fiber quality traits in both environments. Furthermore, 14 SSRs associated with main fiber quality traits in our study coincided with reported fiber quality trait-associated SSRs from QTL-mapping studies in various experimental populations (Tables 11 and 12). At the same time, an additional 86 yet-unreported in literature SSR markers, associated with fiber quality traits in *G*. *barbadense* cotton germplasm, were detected (Table 12).

In a previous study of *G*. *hirsutum* germplasm, 25 fiber quality traits were significantly associated with SSR markers in Uzbekistan and Mexican environments [33]. In analyses of 56 cultivars of *G*. *arboreum* germplasm, 30 fiber trait-associated SSRs were identified [127]. Two independent association-mapping studies of 99 and 241 cultivars from the Chinese *G*. *hirsutum* germplasm collection, revealed 70 and 48 fiber quality trait-associated SSR markers, respectively [19, 80]. Another AM study using 220 cultivars from the US Upland germplasm collection identified 129 fiber trait-associated SSR markers [128]. Notably, several of the identified SSR markers were also reported by previous studies. For example, BNL1521 associated with FL and FS in this study showed the same trait associations in Upland cotton [19]. In previous reports, this marker was also found to be associated with FM and FE [70], FS [79] and FM [21]. Thus, BNL1521 is the high-priority candidate DNA marker for MAS in cotton breeding to improve fiber quality traits.

Association of markers with two or more fiber quality traits indicates the close location of some genes controlling these traits that have been repeatedly observed in many studies [21, 23, 75, 81, 129–131]. Analysis of chromosomal location of identified markers revealed clustering of positively correlated fiber traits on the same chromosome segments (S1 Table). However, two markers were negatively associated with correlated traits (FM-FL and FM-FU). Similar findings were reported by Cai et al. [19] where two markers were associated with FM-FL and FM-FS and were negatively correlated. This suggests the possibility of a joint transfer and inheritance of these traits, thereby bypassing the obstacles in the form of negative correlations.

A fiber traits gene-cluster was identified near markers BNL1421 and BNL1495. The BNL1421, associated with FL in this study, as well as, in a study of *G*. *arboreum* germplasm [71], was associated with FE [78] and FY [72] in *G*. *hirsutum* and located within a chromosome segment that is rich to fiber quality traits [77] (Table 12). The BNL1495, associated with FL in this study, as well as, in a study of *G*. *hirsutum* germplasm [75], was also located within a group of markers associated with FE [78]. Furthermore, an estimated distance between BNL1421 and BNL1495 is ~1,8cM, implying the clustering of fiber quality genes within the selected chromosome segment. BNL1521, located on Ch24 and associated with FL in the current study and in a study of *G*. *hirsutum* germplasm [19], were reported to be associated with FS [19, 79], FM [70, 74] and FE [70]. BNL1705 associated with FL in this study were also reported to be associated with FL [76] and FY [80]. BNL1317 associated with FM herein was also associated with the same fiber trait in other studies [8, 19, 21], FE [19], and FL [70].

Furthermore, BNL1317 was associated with a QTL for phenylalanine content [132], which plays a key role in phenylpropanoid pathway during cotton fiber cell wall formation [133–135]. Thus, BNL1317 is another high-priority candidate marker for MAS. Marker BNL3601, significantly associated with FM (BF ≤0,02) in our study, was also reported to be associated with fiber maturity and fiber cell wall thickness [62], that is directly related to micronaire [136,137]. Therefore, these SSRs should be very useful for fiber quality improvement of cotton cultivars by means of marker-assisted selection (MAS).

In this context, it should be noted that, so far, cotton lags behind on MAS application and success compared to other crops [9,30, 33]. Many molecular markers tagged through numerous traditional QTL-mapping studies, except those monogenically inherited resistance traits (e.g. [138, 139]), have had a limited success in cotton breeding programs [139]. This may be primarily connected with (1) complexity and polyploidy of the cotton genome, (2) polygenic and epigenetic nature of inheritance of many important QTLs including fiber traits, which are greatly impacted from G by E interactions and (3) specificity of tagged molecular markers to a bi-parentally-derived mapping population, making markers meaningless when other populations or genotypes are used [30, 139].

Differing from QTL-mapping approach, LD-based association mapping using germplasm resources helps to associate more biologically meaningful markers in a large number of germplasm accessions, shaped under many historic meiotic events [9, 30, 33, 123]. Therefore, molecular markers associated with important traits using LD-based association mapping should be efficient to be used in MAS programs. For instance, previous efforts on association mapping in a large set of Upland cultivars and exotic landrace stock germplasm [9, 33] have helped us to design a successful molecular breeding program in Uzbekistan. In a short time, using SSR markers associated with fiber length, strength and micronaire traits, novel cotton cultivars series “Ravnaq” (translates from Uzbek as “Advance”) with improved fiber quality traits have been developed, which are currently under evaluation of State Variety Testing Stations of Uzbekistan [139, 140]. This exemplifies the usefulness of genome-wide association mapping studies of cotton that should be highly efficient with application of recently developed genome-anchored SNPs [100] because of genome wide scale and considering many alleles and genetic interactions.

## Conclusions

Thus, in a set of 288 G. *barbadense* germplasm resources from the Uzbekistan cotton collection, for the first time, a genetic diversity, population structure, and the extent of genome-wide linkage disequilibrium were assessed for Pima or extra-long staple cotton genome. Efforts have helped to perform LD-based association mapping of fiber quality traits evaluated in two diverse environments (Uzbekistan and USA) using a highly polymorphic set of simple sequence repeat (SSR) markers. Results have provided important insights into the evolutionary and breeding processes that influence the structure of genetic variation within a population and among populations. Also, there is a lower level of LD compared to the Upland cotton genome or other agricultural crops. Model based-association mapping efforts have further identified strongly associated novel and previously reported SSR markers with major fiber quality traits. Results should help to expand our knowledge of the breeding history and germplasm peculiarities of Pima cotton. Identified SSR markers and candidate gene sequence associated fiber quality traits in *G*. *barbadense* foster cotton improvement programs using marker-assisted selection.

## Supporting information

S1 Data Fiber quality trait data measured for four major fiber traits from Uzbekistan (UZB) and the USA environments. This data set was used for trait analyses and association mapping studies.
(XLSX)

S2 Data TASSEL formatted SSR marker data set for 288 *G*. *barbadense* panel used in this study. Note that 5% MAF is not filtered and can be done using TASSEL.
(XLSX)

S1 Table A set of SSR markers with high polymorphism for studies of the *G*. *barbadense* genome. (DOC)
