# Supplementary material for: Genetic diversity, linkage disequilibrium, and association mapping analyses of Gossypium barbadense L. germplasm
Source: PLoS One. 2017 Nov 14;12(11):e0188125. doi: 10.1371/journal.pone.0188125 (PMC5685624; doi:10.1371/journal.pone.0188125)
Supplement: S1 Table — (DOC) [file pone.0188125.s003.doc]

**S1 Table.** A set of SSR markers with high polymorphism for studies of the *G. barbadense* genome

| # | SSR marker | Polymorphic Information Content (PIC) | Heterozygosity (He) | Chromosome location |
| --- | --- | --- | --- | --- |
| 1 | BNL1317 | 0,41 | 0,50 | A9/D9 |
| 2 | BNL1421 | 0,62 | 0,68 | A13 |
| 3 | BNL1440 | 0,35 | 0,40 | A5/A6 |
| 4 | BNL1495 | 0,62 | 0,68 | A13 |
| 5 | BNL1521 | 0,36 | 0,41 | D8 |
| 6 | BNL1611 | 0,26 | 0,27 | D5 |
| 7 | BNL1667 | 0,61 | 0,67 | A1/D1 |
| 8 | BNL2609 | 0,37 | 0,50 | A5 |
| 9 | BNL2655 | 0,58 | 0,65 | D8 |
| 10 | BNL2872 | 0,37 | 0,50 | A10 |
| 11 | BNL2960 | 0,37 | 0,40 | A10 |
| 12 | BNL3029 | 0,37 | 0,50 | A5 |
| 13 | BNL3065 | 0,48 | 0,57 | D7 |
| 14 | BNL3171 | 0,57 | 0,64 | D11 |
| 15 | BNL3280 | 0,65 | 0,71 | D13 |
| 16 | BNL3398 | 0,34 | 0,40 | A3 |
| 17 | BNL3569 | 0,24 | 0,27 | A5/D5 |
| 18 | BNL3590 | 0,32 | 0,38 | A2/D3 |
| 19 | BNL3601 | 0,32 | 0,41 | D4 |
| 20 | BNL3627 | 0,38 | 0,50 | A8 |
| 21 | BNL3638 | 0,47 | 0,57 | D8 |
| 22 | BNL3792 | 0,36 | 0,39 | A8 |
| 23 | BNL3816 | 0,36 | 0,47 | A12/D12 |
| 24 | BNL387 | 0,33 | 0,42 | A8 |
| 25 | BNL3880 | 0,36 | 0,48 |  |
| 26 | BNL3937 | 0,50 | 0,56 | D6 |
| 27 | BNL3955 | 0,49 | 0,58 | D3 |
| 28 | CM23 | 0,25 | 0,29 | D11 |
| 29 | CM45 | 0,36 | 0,48 | D10 |
| 30 | GH110 | 0,27 | 0,29 |  |
| 31 | GH117 | 0,45 | 0,55 | A4 |
| 32 | GH171 | 0,61 | 0,67 | D8 |
| 33 | GH200 | 0,32 | 0,34 | D4 |
| 34 | GH52 | 0,37 | 0,49 | D4 |
| 35 | GH75 | 0,23 | 0,24 | A1 |
| 36 | GH82 | 0,33 | 0,36 | A6 |
| 37 | JESPR292 | 0,34 | 0,43 | D7 |
| 38 | NAU2913 | 0,39 | 0,46 | D12 |
